# Supplementary material for: Digital contact-tracing during the Covid-19 pandemic: An analysis of newspaper coverage in Germany, Austria, and Switzerland
Source: PLoS One. 2021 Feb 3;16(2):e0246524. doi: 10.1371/journal.pone.0246524 (PMC7857553; doi:10.1371/journal.pone.0246524)
Supplement: S1 Table — (DOCX) [file pone.0246524.s001.docx]

# **S1 News articles included in the analysis**

| No. | Title | Source | Date | Country |
| --- | --- | --- | --- | --- |
| 1 | Gratis-Test bei Benutzung der Covid-App | Tages Anzeiger | 28 May 2020 | Switzerland |
| 2 | Für aktive Unterstützung der Proximity-Tracing-App; Mit weiteren Ausbreitungswellen ist bei der Pandemie zu rechnen. Je schneller Kontaktpersonen isoliert werden können, desto eher lassen sich weitere Ansteckungen verhindern. | Neue Zürcher Zeitung | 28 May 2020 | Switzerland |
| 3 | Suche im virtuellen Heuhaufen; Schon 22 Länder haben mit Tracing-Apps Erfahrungen gesammelt, aber für Deutschland lässt sich daraus nicht viel lernen. Und wenn doch, dann sind die Ergebnisse ernüchternd | Süddeutsche Zeitung | 27 May 2020 | Germany |
| 4 | Corona-App im Google-Store ist keine Panne; Schon 10 000 Mal heruntergeladen | Blick | 27 May 2020 | Switzerland |
| 5 | App könnte das Problem der Veranstaltungen lösen; Fussballspiele mit Zuschauern könnten laut dem Bund bald stattfinden – Kontakte der Besucher müssen aber nachvollziehbar sein | Neue Zürcher Zeitung | 27 May 2020 | Switzerland |
| 6 | Wer in Quarantäne geht, riskiert Lohnausfall | Tages Anzeiger | 26 May 2020 | Switzerland |
| 7 | Pilotphase für Corona-App gestartet; Die offizielle Swiss-Covid-App des Bundes weckt Hoffnungen und Befürchtungen | Neue Zürcher Zeitung | 26 May 2020 | Switzerland |
| 8 | Aufregung um Contact-Tracing für Android | Der Standard | 26 May 2020 | Germany |
| 9 | Gespaltene Meinung zur Corona-App | Bild | 25 May 2020 | Germany |
| 10 | Dann klappt's auch mit dem Handschlag | Die Presse | 17 May 2020 | Austria |
| 11 | Merkels Hirsche und die App | Die Welt | 16 May 2020 | Germany |
| 12 | Wir können dich sehen; Während manche hierzulande von Diktatur faseln, wird unser Korrespondent in China tatsächlich von einer Corona-App umfassend überwacht. Ein Kontrollbericht | Süddeutsche Zeitung | 16 May 2020 | Germany |
| 13 | Indien zwingt Bürger zu Corona-App, die meisten haben kein Smartphone | Kurier | 15 May 2020 | Austria |
| 14 | Corona-App startet nächste Woche mit begrenzter Teilnehmerzahl | Tages Anzeiger | 14 May 2020 | Switzerland |
| 15 | Gute Noten für die Corona-App | Blick | 14 May 2020 | Switzerland |
| 16 | Tracing-App: Aufruf zum Hornberger Schiessen; Eine staatliche Aufspür-App von Corona-Infektionen verspricht weit mehr, als sie einzulösen vermag. Potenzielle Nutzer werden sich gut überlegen, ob sie die App aktivieren werden. | Neue Zürcher Zeitung | 12 May 2020 | Switzerland |
| 17 | «Keine digitale Stasi» | Tages Anzeiger | 11 May 2020 | Switzerland |
| 18 | «Das Virus wartet nicht bis wir fertig getestet haben» | Blick | 11 May 2020 | Switzerland |
| 19 | Nächste Woche startet die Testphase für die Corona-App | Tages Anzeiger | 9 May 2020 | Switzerland |
| 20 | Auf Berset und Parmelin warten knifflige Fragen; Noch ist unklar, was der Bundesrat unternimmt, wenn es zu einer zweiten Corona-Welle kommen sollte | Neue Zürcher Zeitung | 9 May 2020 | Switzerland |
| 21 | App-Benutzer muss man schützen | Tages Anzeiger | 8 May 2020 | Switzerland |
| 22 | App die Plätze, fertig, los!; Verwirrung um Contact Tracing | Blick | 8 May 2020 | Switzerland |
| 23 | Mit Hightech gegen das Virus | Die Welt | 7 May 2020 | Germany |
| 24 | Corona-App muss in einer Pilotphase den Praxistest bestehen | Tages Anzeiger | 7 May 2020 | Switzerland |
| 25 | Ein zweites Mal sind wir viel besser vorbereitet | Blick | 7 May 2020 | Switzerland |
| 26 | Soll man die App installieren? | Blick | 7 May 2020 | Switzerland |
| 27 | Die nächste Nachricht ist vielleicht eine Corona-Warnung | Blick | 7 May 2020 | Switzerland |
| 28 | Jetzt warnt das Handy vor Corona | Blick | 7 May 2020 | Switzerland |
| 29 | Corona-Lockdown: Kritik an Angst und Verwirrung durch die Regierung | Kurier | 5 May 2020 | Austria |
| 30 | Regierung beruhigt: App bleibt freiwillig | Die Presse | 6 May 2020 | Austria |
| 31 | Experiment mit Corona-App auf Isle of Wight | Die Presse | 6 May 2020 | Austria |
| 32 | Doskozil: "Diese Umfrage wollte in der Sozialdemokratie niemand" | Kurier | 5 May 2020 | Austria |
| 33 | FPÖ-Kickl fordert von Kanzler Kurz, Mei-Pochtler "zu entfernen" | Kurier | 5 May 2020 | Austria |
| 34 | And the winner is … | Kurier | 4 May 2020 | Austria |
| 35 | «Die Nutzung einer Tracing-App muss freiwillig sein» | Tages Anzeiger | 5 May 2020 | Switzerland |
| 36 | Ohne Freiwilligkeit gibt’s kein Vertrauen in die Contact-Tracing-App; Ethisch wie politisch scheint weitgehend unbestritten, dass die Nutzung der Contact-Tracing-App freiwillig sein muss. Dies zu gewährleisten, ist jedoch alles andere als trivial. | Neue Zürcher Zeitung | 5 May 2020 | Switzerland |
| 37 | App-Pflicht für alle, die ins Land einreisen? | Die Presse | 5 May 2020 | Austria |
| 38 | Kanzlerberaterin am Rande | Der Standard | 5 May 2020 | Germany |
| 39 | Corona-App gibt mehr Freiheiten | Die Welt | 4 May 2020 | Germany |
| 40 | Kurz-Beraterin zu Contact-Tracing-App: "Wird Teil der neuen Normalität" | Kurier | 4 May 2020 | Austria |
| 41 | Datenschutz ist kein Gesundheitsrisiko | Der Standard | 4 May 2020 | Germany |
| 42 | Holodeck: Corona-App. Die Diskussion um die App des Roten Kreuzes ist fadenscheinig. Anstatt uns mit Babyelefanten zu nerven, sollte uns die Regierung die Corona-App erklären. Sauber, ordentlich und glaubwürdig | Die Presse | 3 May 2020 | Austria |
| 43 | CORONA-APP; Zwischen Nutzen und Schaden | Süddeutsche Zeitung | 2 May 2020 | Germany |
| 44 | Nur jeder zweite Parlamentarier will die App installieren; Experten preisen Tracing-App als wichtiges Mittel zur Eindämmung der Pandemie – doch bei den National- und Ständeräten gibt es viele Skeptiker | Neue Zürcher Zeitung | 2 May 2020 | Switzerland |
| 45 | Was in den nächsten Monaten gelingen muss | Die Presse | 2 May 2020 | Austria |
| 46 | App soll Massen-Ansteckungen verhindern | Blick | 30 April 2020 | Switzerland |
| 47 | Wirtschaft Kompakt: Corona-App: Telekom und SAP als Entwickler an Bord | Die Welt | 29 April 2020 | Germany |
| 48 | Unser Verlangen nach starker Führung | Tages Anzeiger | 29 April 2020 | Switzerland |
| 49 | Contact-Tracing: Das Vertrauen nicht verspielen; Drei Aspekte haben bisher zu wenig Beachtung gefunden: Erfahrungen aus dem Public-Health-Bereich, der rechtliche Rahmen sowie die Rolle ethischer Prinzipien | Neue Zürcher Zeitung | 29 April 2020 | Switzerland |
| 50 | «Für die Contact-Tracing-App braucht es keine Freiwilligkeit»; Der Zürcher Datenschützer sieht kein Problem bei einem geeigneten und zur Eindämmung der Corona-Pandemie erforderlichen Instrument | Neue Zürcher Zeitung | 29 April 2020 | Switzerland |
| 51 | Nur die zweitbeste App | Die Welt | 28 April 2020 | Germany |
| 52 | Nationalrat: Neue Vorschriften für Demos | Kurier | 28 April 2020 | Austria |
| 53 | Nadeln sammeln statt Heuhaufen; Durch übertriebene Überwachung hat der Staat viel Vertrauen verspielt. Die geplante Corona-App dagegen könnte zeigen, dass man die Epidemie bekämpfen kann, ohne Bürgerrechte zu missachten | Süddeutsche Zeitung | 28 April 2020 | Germany |
| 54 | Tagebuch der Kontakte; Sie soll den Datenschutz gewährleisten und möglichst schnell verfügbar sein: die wichtigsten Antworten zur geplanten Corona-App | Süddeutsche Zeitung | 28 April 2020 | Germany |
| 55 | Kritische Masse | Süddeutsche Zeitung | 28 April 2020 | Germany |
| 56 | Erst das Tracking,- dann die Moral | Die Presse | 28 April 2020 | Austria |
| 57 | Ist eine App-Pflicht rechtlich möglich? | Die Presse | 28 April 2020 | Austria |
| 58 | Wie wirken Apps & Co. global? | Die Presse | 28 April 2020 | Austria |
| 59 | Wie sicher sind die Daten der User? | Die Presse | 28 April 2020 | Austria |
| 60 | Was plant die heimische Politik? | Die Presse | 28 April 2020 | Austria |
| 61 | Wenn das gute Gewissen- den eigenen Vorteil sticht | Die Presse | 28 April 2020 | Austria |
| 62 | Kann eine App gegen Corona helfen? | Die Presse | 28 April 2020 | Austria |
| 63 | Was machen Google und Apple? | Die Presse | 28 April 2020 | Austria |
| 64 | Skandinavier gehen unterschiedliche Wege; Norwegen nutzt Tracking-App bereits – Schweden arbeitet vorerst mit Website | Der Standard | 28 April 2020 | Germany |
| 65 | Israelisches Gericht stoppt App; Österreichische Grüne weiterhin gegen Tracking-Apps | Der Standard | 28 April 2020 | Germany |
| 66 | Regierung setzt nun auf dezentrale Corona-App | Die Welt | 27 April 2020 | Germany |
| 67 | Regierung einigt sich auf Tracing-App; Gesundheitsminister und Kanzleramt sprechen sich für eine Anti-Corona-Anwendung aus, die Kontakte dezentral speichert. Die Software müsse bald zur Verfügung stehen, fordert die Opposition | Süddeutsche Zeitung | 27 April 2020 | Germany |
| 68 | Corona-App soll kein Eventticket werden | Der Standard | 27 April 2020 | Germany |
| 69 | Türkis-Grün will niemanden von Events aussperren; Klubobleute Wöginger und Maurer: Keine indirekte Pflicht zur Corona-App | Der Standard | 27 April 2020 | Germany |
| 70 | Vorbild Singapur: Austrialen führt Corona-App ein | Kurier | 26 April 2020 | Austria |
| 71 | Entscheidung über Corona-App liegt bei Anschober | Kurier | 26 April 2020 | Austria |
| 72 | Deutschland will Corona-App für "möglichst breite Teile der Bevölkerung" | Kurier | 26 April 2020 | Austria |
| 73 | Die gescheiterte App-Politik | Die Presse | 26 April 2020 | Austria |
| 74 | Coronavirus: Pensionistenverband warnt vor "Wegsperren" der Senioren | Kurier | 25 April 2020 | Austria |
| 75 | Suche nach Infizierten geht weiter - vielleicht auch mit freiwillig genutzter App | Tages Anzeiger | 25 April 2020 | Switzerland |
| 76 | Opposition befürchtet Pflicht für Corona-App; Gesetz soll Hintertüre bieten, Minister Anschober kalmiert | Der Standard | 25 April 2020 | Germany |
| 77 | "Wir brauchen eine Armee von Contact Tracern" | Die Welt | 24 April 2020 | Germany |
| 78 | Ohne Vertrauen scheitert die Corona-App; Contact-Tracing | Neue Zürcher Zeitung | 24 April 2020 | Switzerland |
| 79 | Gutes Zeugnis für "Stopp Corona" | Der Standard | 23 April 2020 | Germany |
| 80 | Der virologische Imperativ | Die Welt | 23 April 2020 | Germany |
| 81 | Wird die Corona-App zur Pflicht?; Bedenken wegen Datenschutz | Blick | 23 April 2020 | Switzerland |
| 82 | Die Corona-App ermächtigt uns | Tages Anzeiger | 23 April 2020 | Switzerland |
| 83 | Bund könnte Corona-App vorschreiben | Tages Anzeiger | 23 April 2020 | Switzerland |
| 84 | "Corona-App" besteht Prüfung | Die Presse | 23 April 2020 | Austria |
| 85 | Die App, die bei einer Ansteckung warnt | Tages Anzeiger | 22 April 2020 | Switzerland |
| 86 | Corona-Tracing-App soll im Mai in der Schweiz starten; Die offizielle App des Bundes könnte helfen, Infektionsketten zu unterbrechen – andere europäische Länder sind noch nicht so weit | Neue Zürcher Zeitung | 22 April 2020 | Switzerland |
| 87 | Starke Zweifel an Corona-App; Experten warnen vor Mängeln beim Datenschutz | Süddeutsche Zeitung | 21 April 2020 | Germany |
| 88 | Corona-App droht zu scheitern | Die Welt | 23 April 2020 | Germany |
| 89 | Streit um App für Corona-Tracing; Schweizer Forscher verlässt paneuropäisches Projekt | Neue Zürcher Zeitung | 18 April 2020 | Switzerland |
| 90 | Salathé steigt aus Projekt für Corona-App aus | Tages Anzeiger | 18 April 2020 | Switzerland |
| 91 | Das Vertrauen der Menschen ist entscheidend; Die geplanten EU-weit kompatiblen Nachverfolgungs-Apps müssen die Privatsphäre und die Daten der Bürger schützen | Neue Zürcher Zeitung | 17 April 2020 | Switzerland |
| 92 | App müsste von Millionen geladen werden; Das BAG will das Smartphone zur Bekämpfung der Corona-Pandemie einsetzen, doch das ist gar nicht so einfach | Neue Zürcher Zeitung | 16 April 2020 | Switzerland |
| 93 | Nur die staatliche App schafft Vertrauen; Contact-Tracing mittels einer Smartphone-App verlangt transparente Rahmenbedingungen. Dies kann nur der Staat gewährleisten. | Neue Zürcher Zeitung | 16 April 2020 | Switzerland |
| 94 | Corona-App: FPÖ bringt Anzeige bei Datenschutzbehörde ein | Kurier | 15 April 2020 | Austria |
| 95 | Die Corona-App mit (wenig) Handschlagqualität | Die Presse | 16 April 2020 | Austria |
| 96 | Corona-Apps könnten an Nutzermangel scheitern | Die Welt | 15 April 2020 | Germany |
| 97 | «Es ist der richtige Zeitpunkt» | Tages Anzeiger | 15 April 2020 | Switzerland |
| 98 | Wie Apps das (EU-weite) Reisen erleichtern sollen | Die Presse | 15 April 2020 | Austria |
| 99 | Deutschlands Außenminister für einheitliche Corona-App in der EU | Kurier | 14 April 2020 | Austria |
| 100 | Das Smartphone kann aus dem Stillstand führen; Auch in der Schweiz wird nach Ortungsmethoden gesucht, um die Corona-Krise zu überwinden. Das Smartphone ist aber in erster Linie ein Instrument der Selbstbefähigung. Zu einem Gängelband des Staates darf es nicht werden. | Neue Zürcher Zeitung | 14 April 2020 | Switzerland |
| 101 | Apple und Google spannen in der Corona-Krise zusammen; Bereits in einem Monat soll eine neue Technologie für das Contact-Tracing zur Verfügung stehen | Neue Zürcher Zeitung | 11 April 2020 | Switzerland |
| 102 | Apple und Google gegen Covid-19; Tech-Rivalen bauen Software für Contact-Tracing | Neue Zürcher Zeitung | 11 April 2020 | Switzerland |
| 103 | Eine Covid19-Boardingcard für Lokal- und Barbesuche | Die Presse | 11 April 2020 | Austria |
| 104 | WKO-Präsident Mahrer: "Reden wir endlich über Herden-Kreativität" | Kurier | 10 April 2020 | Austria |
| 105 | Wie der Alltag mit der App aussehen wird | Die Presse | 11 April 2020 | Austria |
| 106 | Wie eine zweite Welle verhindert wird | Die Presse | 11 April 2020 | Austria |
| 107 | "Stopp Corona"-App trackt automatisiert; Neue Version und frische Daten von A1 | Der Standard | 11 April 2020 | Germany |
| 108 | Kein Allheilmittel, aber gut | Der Standard | 10 April 2020 | Germany |
| 109 | Eine Frage des Vertrauens | Der Standard | 10 April 2020 | Germany |
| 110 | Hat man die Corona-App, klingelt jeden Tag die Polizei | Die Welt | 9 April 2020 | Germany |
| 111 | Anschober: "Müssen Zeit gewinnen, bis es einen Impfstoff gibt" | Kurier | 8 April 2020 | Austria |
| 112 | "Stopp Corona" erhält neue Funktionen | Der Standard | 9 April 2020 | Germany |
| 113 | RKI sammelt mit App Corona-Daten | Bild | 8 April 2020 | Germany |
| 114 | Corona-App wird digital aufgerüstet | Die Presse | 8 April 2020 | Austria |
| 115 | «Ich sehe die Gefahr, dass sich autoritäre Systeme etablieren» | Die Presse | 8 April 2020 | Austria |
| 116 | Erste Massnahmen gegen die zweite Welle | Blick | 7 April 2020 | Switzerland |
| 117 | Schöne neue Welt; Die Analyse von Handy- und Verbraucherdaten kann helfen, die Verbreitung des Corona-Virus einzudämmen. Privatsphäre und Gemeinwohl, Zwang und Freiwilligkeit - Staaten müssen nun schwierige Abwägungen treffen | Süddeutsche Zeitung | 7 April 2020 | Germany |
| 118 | Österreich debattiert über verpflichtende Corona-App; Das Land nutzt bereits eine vom Roten Kreuz entwickelte Software, die nun ausgebaut werden soll | Süddeutsche Zeitung | 6 April 2020 | Germany |
| 119 | Kurz plant Lockerung der Beschränkungen | Tages Anzeiger | 6 April 2020 | Switzerland |
| 120 | Ein freiwilliges Kontakt-Tagebuch | Die Presse | 6 April 2020 | Austria |
| 121 | Kurz will "Versuch eines Fahrplans" präsentieren | Der Standard | 6 April 2020 | Germany |
| 122 | Sobotka blitzte mit Forderung nach verpflichtender App ab; Nach scharfer Kritik setzt der Nationalratspräsident nun auf "Freiwilligkeit" | Der Standard | 6 April 2020 | Germany |
| 123 | Wachsam bleiben | Der Standard | 6 April 2020 | Germany |
| 124 | Sobotka rudert zurück: "Wir setzen bei Stopp-Corona-App auf Freiwilligkeit" | Kurier | 5 April 2020 | Austria |
| 125 | Transparenz ist den Menschen zumutbar | Die Presse | 5 April 2020 | Austria |
| 126 | Wieder raus mit Maske und Handy-App | Die Presse | 5 April 2020 | Austria |
| 127 | Mit Smartphone-Daten gegen das Virus; Asiatische Länder gehen bei der Auswertung sehr weit – das weckt Begehrlichkeiten auch in Europa | Neue Zürcher Zeitung | 4 April 2020 | Switzerland |
| 128 | Viren-Tracking: Jetzt kommt Chip auf den Schlüsselbund oder HandyApp | Kurier | 4 April 2020 | Austria |
| 129 | Sobotka für verpflichtende Corona-App | Kurier | 4 April 2020 | Austria |
| 130 | Der diskrete Financier der Corona-App | Die Presse | 4 April 2020 | Austria |
| 131 | App soll Seuche stoppen | Blick | 3 April 2020 | Switzerland |
| 132 | Max Schrems: "So macht die Corona-App wenig Sinn" | Kurier | 3 April 2020 | Austria |
| 133 | Eine Smartphone-App könnte das Virus stoppen; Eine Software-Lösung beschleunigt die Nachverfolgung der Kontakte von Covid-19-Infizierten – und trägt dem Datenschutz Rechnung | Neue Zürcher Zeitung | 3 April 2020 | Switzerland |
| 134 | Schweiz beteiligt sich an europäischer App gegen das Virus | Tages Anzeiger | 2 April 2020 | Switzerland |
| 135 | Eine App soll das Leben wieder normaler machen | Die Welt | 2 April 2020 | Germany |
| 136 | So funktioniert das neue Smartphone-Warnsystem | Die Welt | 2 April 2020 | Germany |
| 137 | Corona-Stopp-App entwickelt | Süddeutsche Zeitung | 2 April 2020 | Germany |
| 138 | Tut mir leid, aber- wir müssen reden | Die Presse | 2 April 2020 | Austria |
| 139 | Mit Smartphone-Apps dem Coronavirus auf die Spur kommen; Programmierer haben eine Software entwickelt, die Kontakte mit infizierten Menschen nachzeichnen kann – die Privatsphäre wird dabei nicht verletzt | Neue Zürcher Zeitung | 1 April 2020 | Switzerland |
| 140 | Stopp Corona"-App: Mehr als 130.000 Nutzer in der ersten Woche | Kurier | 1 April 2020 | Austria |
| 141 | «Kein Fall darf vernachlässigt werden»; Der Epidemiologe Marcel Salathé schlägt eine Strategie vor, wie die Schweiz aus dem Lockdown herauskommen könnte | Neue Zürcher Zeitung | 31 March 2020 | Switzerland |
| 142 | Staatliche Tracking-App bei Bedarf denkbar | Die Presse | 30 March 2020 | Austria |
| 143 | Mögliche Wege zurück in die Normalität; Der gesellschaftliche Notbetrieb kostet jede Woche Milliarden – wie könnte man den Stillstand überwinden? | Neue Zürcher Zeitung | 28 March 2020 | Switzerland |
| 144 | Der große Bruder in der Tasche | Der Standard | 28 March 2020 | Germany |
| 145 | Rotes Kreuz startete "Stopp Corona"-App | Kurier | 25 March 2020 | Austria |
| 146 | Stopp Corona App: "Sorgen uns nicht um Daten-, sondern um Menschenschutz" | Kurier | 24 March 2020 | Austria |
| 147 | Spahn verliert (vorerst) Kampf ums Smartphone | Die Presse | 24 March 2020 | Austria |
| 148 | Das Handy als Virendetektor | Tages Anzeiger | 19 March 2020 | Switzerland |
